# A New System of Organic Chemistry

**Published:** 1834-10-01

**Authors:** 


A New System of Organic Chemistry. By *F. V. Raspail.* Translated by *W. Henderson,* M.D. Octavo, pp. 602. London, 1834.

This is one of the most ingenious and original works which we have perused for a long time. M. Raspail is evidently a man of superior talent, and this talent is happily associated with the most unwearied zeal and assiduity: his imagination, indeed, like that of most of his countrymen, is apt to be too vivacious, and the celerity of many of his conclusions may somewhat exceed the credulity of a reader on this side of the Channel: but, barring this, every one who peruses the present work must feel pleasure in awarding to M. Raspail the praise of being a most clever chemist, and a subtle and original thinker. From his own account, we are led to suppose that he has been ill used, and that his countrymen, and more especially the members of the French Institute, have been for many years his sworn enemies, and tried in every way to rob him of his fair deserts. We have seen it stated somewhere, that all his devotedness to chemical pursuits had not been able to keep him out of the broil of political agitation, and that, whether justly or unjustly we cannot tell, his one-sided sins had been visited by the public authorities with "durance vile." He confesses, indeed, the want of patronage and the want of funds in the prosecution of his labours; but these seem only to have redoubled his enthusiasm, and many a humble student of our classes and colleges may be inspirited by our author's appeal, when he tells him "not to be discouraged, when his fortune does not permit him to realize the plan which has at first presented itself to his mind. How often, after having cursed my poverty, and despaired of the success of my trials for want of money, has it happened to me that I have, by a sudden thought, conceived the

---

idea of an article not worth a penny, which served every purpose that could have been answered by a more expensive apparatus."

This work is entitled a "New System of Organic Chemistry;" *new*, not because it is the most recent, but because it is constructed on different principles, and is developed in a different manner, from what has been attempted by preceding authors. M. Raspail very justly remarks, that chemists have hitherto taken but a very limited, and, therefore, an unscientific view of the composition of organic substances, and he traces most of their errors to their attention having been confined to the mere results of analysis in the laboratory, without appealing to other sources of information. The crucible, the test-tube, the blow-pipe, &c. may be all quite necessary to such an enquiry, but they ought not to be the only tools we work with; and before, indeed, we have recourse to so rude and destructive weapons, our author very aptly exhorts the use of others, which are alike more easy of employ, and often as potently useful. By far the most important of these is the microscope, and, with its wonderful aid, we shall find that our researches are at once made both more amusing and instructive. Had M. Raspail no other claim to our approbation, save that of having introduced this instrument as an essential constituent of the chemist's apparatus, he would have deserved well of science; for although others, among whom we record with especial praise our eminent countrymen, Wollaston, Young, and Brewster, had previously employed it in their enquiries, yet Dr. Henderson is quite justified in asserting that M. R. has "made a more universal application of it to organic chemistry than had before been done, by any one who was able to avail himself of the more accurate principles of chemical science that have been established by the enquiries and discoveries of late years."

There is so much truth, so well expressed, in the following observations, that we cannot deny ourselves the pleasure of extracting them; and their value is enhanced by the consideration, that the spirit which they breathe ought to animate, not only the philosophic chemist, but also the physiologist, the botanist, and natural historian.

"Now Nature is not to be considered as either a Chemist, or a Botanist, or a Zoologist, or a Mineralogist, or a Physiologist. It is not divided into scientific compartments. It does not proceed according to classifications and artificial systems. It is a single cause producing varied combinations.

It is, therefore, absurd to study these combinations only in one view; and yet this is almost precisely what the different sciences have done to this day; and this is the cause of a multitude of errors and misconceptions, and of the dispiriting slowness of the progress of scientific research.

We must, therefore, have recourse to a more rational method—to a more philosophical way of proceeding, if we would arrive at more positive results.

Now this new method may be summed up in these terms:—To borrow from each science all that may be useful for ascertaining a fact, or establishing a law; for, although a book may very properly be special, being a collection of a certain order of facts, an Observer, who confines himself within the circle of a speciality is either incapable or inconsequent.

Certain substances being by natural processes deposited within certain organs, I shall appeal to Anatomy for the means of recognising these organs; and, when once I have learned to distinguish them, I shall apply to Chemistry for the information which its reactions and its processes can furnish. If these organs are too small to be seen by the eye simply, I shall employ the assistance of the magnifying-glasses of the microscope. Physical science will teach me to follow the course of the luminous rays, and will give me a knowledge of the effects of refracted and reflected light; and I shall establish a chemical laboratory on the object-holder of the microscope." xv.

It would be quite inconsistent with the general scope of this review, devoted as it is to the more immediately practical departments of professional inquiries, to enter upon an examination of this work in detail; but in our capacity as supervisors of medical literature, we feel it a duty to draw the attention of all who are interested in the delightful pursuits of chemical science to the "new system" of M. Raspail, as a work admirable alike for the ingenuity of its speculations, and for the mass of instructive material contained in its pages. A chemist would do well to con over, and try by the test of direct experiment almost every proposition as he goes along. We should be prepared indeed to hear that he dissented from many of their data and inferences, but we shrewdly suspect, that the very refutation of these would lead him to some very important and unexpected results; for there is the germ of genius pervading almost every portion of the work.

We shall content ourselves with selecting certain passages which either apply more immediately to medicine, or are calculated to enlighten the general reader.

At page 176 our author states—

"It has been observed that cotton cloth, however fine, cannot, without injury, be substituted for linen in the preparation of surgeon's lint; and some authors have imagined that they had found the reason of this, in the shape of the fibres of cotton, which, according to them, are triangular, and with sharp angles capable of cutting and irritating the flesh. This explanation, which at best is ridiculous, has been admitted by authors of note in chemistry, who are in general sufficiently difficult on the explanation of facts. Yet it was very easy to see that so minute organs, even if they were as sharp as was supposed, could do little harm, when separated from the living flesh by an inert coagulum. Besides, the microscope assures us that this shape of the fibres of cotton exists only in the imagination of those who have not observed them. The fibres of cotton are tubes analogous to the small hairs of the *Gramina* of which I have spoken, although much larger; they become flat by drying, after which they present the appearance of a band with fringed edges and a raised border. An enlarged figure of them was given in a small essay which I published in 1827 in the *Bibliotheque Physico-Economique*. Finally, it is certain also, that these bands or fibres of cotton are much more flexible than the tubes of hemp or of flax. If, then, the lint acted mechanically, that which is made of hemp or flax ought to be more hurtful than that which consists of cotton; and yet experience shows the contrary. We must then seek the explanation elsewhere, and a very natural one is to be found in the phenomena of capillary attraction. The fibres of hemp and flax are tubes open at both ends, and the watering to which they had been subjected has emptied them of all the juices which they contained. Those of cotton, on the other hand, are hairs shut at both ends, and filled with a *substance tending to organize*, which no watering or washing can remove from them. It is, then, evident that the tubes of linen will be more proper than the hairs of cotton for imbibing blood or pus; for lint made of the latter will not imbibe any thing, but will only allow a free passage among its fibres to any liquid which would have run off just in the same manner without it." 177.

On the curious subject of "muscular contraction," M. Raspail adverts to the doctrine of those who maintain that the fibres of a muscle, when it contracts, assume a zig-zag direction. Prevost and Dumas have lately adopted this opinion, and endeavoured to demonstrate its truth, by an appeal to certain electro-microscopic observations. Having placed a lamina of muscular tissue in the focus of the microscope, and exposed it to galvanic action, they perceived, we are told, each fibre bend into a zig-zag, forming angles whose apices coincided with the terminations of the nervous filaments. The objections which our author urges against these statements are forcibly conveyed in the following passage :—

" 1st, It is difficult to conceive how elastic filaments could form themselves into lines so sharply angled as those figured by the authors of this essay. 2d, They ought to have taught us how to distinguish the muscular fibres from the ultimate filaments of the nervous system. When once the nerves have diminished to the size of the elementary cylinders of muscular fibre, I declare for my part, that it would be impossible for me to distinguish by the microscope what belongs to the nerve and what to the muscle. Anatomists know well that, in following by the lens the nerves to their ultimate ramifications, it becomes very difficult for them to decide on the nature of the texture which they observe; how difficult, then, must it be with the microscope, where very often the eye alone is appealed to, and where the scalpel cannot be employed to trace and unravel the fibres? 3d, Even if these authors did see something analogous to the figures which accompany their descriptions, this experiment would in no way prove what they advance. The muscular lamina is, in fact, necessarily in contact with the object-holder in several points; and, hence, if any tremor be excited in it, either mechanically or by applying the galvanic power to the nervous fibre, this tremor will of itself be sufficient (from the resistance given by the points adhering to the object-holder) to cause sinuous movements, which afterwards in designing the figures have been rendered more or less regular and more or less angular. The result of the observation is then altogether artificial, and cannot in any way be considered as representing what takes place in nature." 261.

M. R. very properly prefers making the experiment, when a muscle contracts under the influence of the vital force, or of its ordinary stimulus, and assures us that he has repeatedly examined the moving organs of many of the lower animals, such as the foot in the anodontes, gasteropodes, &c. and that he has invariably found that the "contractions took place only by means of a shortening of the muscular fibres, and that this shortening was accompanied by an increase in their diameters, which caused small swellings throughout their whole extent."

The next subject which we shall select for notice is one which cannot fail to interest the physiological reader, and as the phenomena which we propose describing briefly, have not been alluded to in any of our medical journals, the account of them will possess all the freshness of novelty to our readers. It is now some years ago since Corti in Italy announced that if a portion of the "chara hispida" (a cryptogamic plant abounding in our marshes and ponds) be examined under the microscope, a most beautifully distinct circulation of globules may be perceived in each internodium or interval, between the transverse septa, which are seen to divide it into numerous segments or compartments.

The truth of this discovery may be most easily verified by any one, as the plant is most common, and we do not require a powerful microscope for the purpose.

M. Raspail has for the last two years performed a multitude of experiments, and the details of some of those we now propose to communicate.

"Let an internodium of the *Chara Hispida* be detached from the stem by cutting it off beyond the articulations that terminate it at each end, taking care to remove all the verticillated branches. The bark which covers it is to be taken off in the following manner :—The internodium is stretched on a glass plate, whose length is less than the distance between the two articulations, and which is placed in a shallow capsule full of water. Each of the small cylinders of the bark is to be raised with the point of a scalpel, and (taking care not to go too deep) the scalpel is to be carried from one end of the internodium to the other, so as to detach them entirely from the trunk. When all the cylindrical thongs of bark are removed, there is brought into view a thick cylinder incrusted with a white substance which adheres strongly to it and is hard and brittle resisting the edge of the scalpel and assuming a farinaceous appearance on drying. This substance is carbonate of lime, which must be removed with a blunt knife by scraping the tube lengthways, holding the blade perpendicularly. The tube being thus prepared, it is immersed in water and placed in the focus of the microscope. The following phenomena may then be observed :—

Through the transparent sides of the tube two opposite longitudinal currents may be seen. They appear to be separated by a longitudinal line, which may be seen on the opposite sides of the tube, and which is distinguished by its whiteness and transparence from the green and granulated layer that lines the inside of the tube. Each of these currents carries with it globules or clots of different sizes, which show its course, but which never mix with those of the opposite current. Sometimes, however, there are seen, on the line of separation, large globules of a more or less cellular structure, which, being kept at the bottom of the liquid by their specific gravity, are there subjected to the resultant of the two simultaneous and opposite forces of the two opposite currents, and consequently turn round on their axis." 357.

A curious phenomenon connected with this vegetable circulation was discovered by Gozzi, and has been repeatedly verified and illustrated by M. Raspail. If ligatures be put round a portion of the tube, at a short distance from each other, and between any two articulations, and the tube be then cut across, between these and the ligatures (so that we have a tube with factitious articulations) not only will the circulation be found to continue, but after a few days the ligatures fall off, and the ends of the tube remain completely closed by the spontaneous adherence of their sides.

"An artificial tube thus prepared is well adapted for the purpose of observing all the phenomena of the circulation. We see, in fact, that the current when it reaches one of the extremities of the tube, makes the circuit of the round end produced by the adherence of the sides, and immediately assumes the opposite direction." 357.

We are told by our author that there is no partition between the two currents, and he has given directions how to ascertain this point. The smallest interruption of the continuity of the green membrane which lines the tube is sufficient to stop the circulation ; or if it still continue for a few moments, it will be seen that the circulating fluid avoids the spot from which the green matter has been removed, and that generally nothing passes across this white spot. The integrity of the green membrane seems therefore indispensable to the existence of the circulation. Accordingly, if we bend the tube in the smallest degree we shall certainly arrest the circulation in its interior. Another condition essential to the continuance of the circulation is the moisture of the plant, and hence—

"If we place a tube peeled and deprived of its incrustation in the focus of the microscope, moistening it with a small drop of water, we shall see that as the water evaporates the internal motion becomes slower; but, if, when it is just about to stop entirely, we apply a drop of water to any point of the tube, we shall immediately see the portion of the internal liquid adjacent to this moistened part of the tube start as it were and begin to move; and, if we then spread the drop of water with a straw over the rest of the tube, the circulation will be re-established in all its former regularity." 360.

The circulation is immediately arrested when a drop of alcohol, liquid ammonia, of pure fixed alkali, or of acid, either vegetable or animal, is placed on the surface of a peeled tube. (There are several engravings at the end of the volume which very beautifully illustrate many of the preceding statements respecting the circulation in the chara.) To attempt to explain the cause of the circulation, which we have now described, on mere hydraulic principles, as M. Raspail has done, appears to us most unsatisfactory and irrational. The French school of physiologists is, as we have frequently of late taken occasion to notice, fast verging to all the presumptuous decisiveness of cold Materialism, such as characterized their countrymen half a century ago. The last editions of Richerand's and of Majendie's systems of Physiology are every where pervaded with this most unphilosophic spirit; but we are rejoiced to think that it has not yet diffused itself beyond the boundaries of "la belle France," and sincerely happy are we, that by far the boldest as well as the most talented disciple of the Materialist school, on this side of the Channel, has within this very present year, and in this very metropolis, publicly yet spontaneously recanted the errors he had too rashly advocated, and has brought himself to acknowledge, that the operations of living bodies are not to be explained on the principles of mere physical influences, and that it is wiser to confess our ignorance than daringly to challenge the wisdom and goodness of an Almighty power.

The comprehensive scope of M. Raspail's work leads him in different parts of it to allude to some of the highly important subjects which may become the themes of examination and disputes in Courts of Law, and the right decision of which may reflect so much credit and honor on the testimony of the medical witnesses. Well indeed does our author say that—

"A mistake may be corrected in chemistry, but in legal medicine it is irreparable. The sword of the law does not retrace its steps as the opinion of a medical man may; and, for this reason, I have never since 1828 allowed any opportunity to escape of rebuking the temerity with which medico-legal opinions are generally given before the judge." 416.

These few lines are introductory to some valuable remarks which M. R. makes on the question "whether it might be ascertained, to what class of animals blood which had become dry, and had produced a given spot, belonged, by simple inspection of its globules, or even by a chemical examination of them." The affirmative was maintained by a chemist of celebrity in Paris some years ago, but he was soon compelled to renounce this opinion, in consequence of the cogent objections which our author adduced soon after.

"About the same time," we are told, "Orfila, relying on experiments on the large scale, announced, in an essay of considerable length, that a spot of blood may be distinguished from a red spot produced by any other substance. I combated this opinion; and, to prove its incorrectness by experiment, I brought forward spots made with the albumen of a pullet's egg in which I had steeped a small bag filled with *madder* slightly moistened. The effect of the reagents mentioned by Orfila was precisely the same on these spots as on real spots of blood. In a succeeding essay Orfila pointed out a difference between the spots of blood and these artificial spots, viz.—that after being boiled the spot of blood appeared greenish by reflection, while the colour of the artificial spot remained unchanged. I replied, that he had gained nothing by this, inasmuch as the object was not to ascertain whether the artificial spots were identical with the real ones or not—but only to prove how deceptious the reagents mentioned in his essay were, as applied to a substance of so complicated a nature as blood, since he had found it necessary to renew his inquiry and have recourse to new criteria to distinguish a real spot of blood from another spot of a kind not previously compared with it; and that it was possible to make a new mixture which this new test would not be able to detect, by adding to the spot of albumen and madder a portion of tannin and of a salt of iron, in such a manner that they should not act on each other till they were boiled together. I added, that nature abounds in mixtures whose study the chemist has not yet entered on, and which might be capable of presenting, on a small scale, the appearance of blood, since this liquid is nothing but a mixture of albumen, dissolved and undissolved, with various salts, a ferruginous colouring matter, and water—substances which might be brought together naturally or accidentally in twenty different ways.

The dispute was keen, as is always the case on medical subjects; but the alarm was given, doubts began to be entertained, and, finally the opinion was abandoned." 417.

From the preceding extracts our readers may judge of the valuable contents of the work now under review.

